# Economic Burden of Treatment‐Resistant Depression Among Patients Hospitalized for Major Depressive Disorder in the United States

**DOI:** 10.1176/appi.prcp.20190001

**Published:** 2019-10-11

**Authors:** Jay Lin, Holly Szukis, John J. Sheehan, Larry Alphs, Brandy Menges, Melissa Lingohr‐Smith, Carmela Benson

**Affiliations:** ^1^ Novosys Health Green Brook New Jersey; ^2^ Janssen Scientific Affairs Titusville New Jersey

**Keywords:** Major depressive disorder, Treatment‐resistant depression, Hospital resource use, Hospital costs, Readmission risk

## Abstract

**Objectives:**

This study aimed to evaluate hospital length of stay (LOS) and cost as well as readmission risk and the associated economic burden among patients hospitalized for treatment‐resistant and non–treatment‐resistant major depressive disorder.

**Methods:**

Adult patients with a primary hospital discharge diagnosis of major depressive disorder were identified from the Premier Hospital Database (January 1, 2012–September 30, 2015). Patients were stratified into two cohorts: those whose hospital treatment was suggestive of treatment‐resistant depression and those with non–treatment‐resistant depression. Hospital LOS and cost during the initial admission and readmissions rates, LOS, and cost within the 6‐month follow‐up were compared between cohorts with a propensity score–matched sample.

**Results:**

After matching, 45,066 patients were included in each cohort. For index hospitalizations, mean hospital LOS was longer (7.4 vs. 5.9 days, p<0.001) and mean hospital cost higher ($8,681 vs. $6,632, p<0.001) for patients with treatment‐resistant depression vs. non–treatment‐resistant depression. Rates for all‐cause (24.4% vs. 20.0%, p<0.001), major depressive disorder–related (17.0% vs. 13.3%, p<0.001), and suicidal ideation/suicide attempt–related (12.8% vs. 9.5%, p<0.001) readmissions were higher for patients with treatment‐resistant depression. Mean LOS and total hospital costs per patient for readmissions were also greater for patients with treatment‐resistant depression vs. non–treatment‐resistant depression. Correspondingly, the combined hospital cost (index hospitalization+all‐cause readmissions) was greater for patients with treatment‐resistant depression ($12,370 vs. $9,429, p<0.001).

**Conclusions:**

Treatment‐resistant depression was associated with substantial economic burden among patients hospitalized for major depressive disorder. More‐effective treatment and care for this patient population may reduce the hospital burden of patients with treatment‐resistant depression.

Major depressive disorder is a chronic, debilitating condition associated with depressed mood, impaired function, suicidal behavior, and frequent health care resource utilization. According to a national study of U.S. adults, the 12‐month prevalence of major depressive disorder is 10.4%, with 39% of episodes classified as severe (1). Numerous established pharmacologic monotherapies and combination therapies, depression‐focused psychotherapies, and device‐based therapies are available for treatment of major depressive disorder. However, many patients who receive treatment and continue to experience persistent symptoms are categorized as having treatment‐resistant depression (2, 3). The Agency for Healthcare Research and Quality (AHRQ), in a summary statement of the various definitions of treatment‐resistant depression, states that patients with this condition are most commonly defined as those who fail to respond or enter remission after two or more antidepressant treatments provided at adequate dosages for adequate durations (4). However, the AHRQ also states that the definition of treatment‐resistant depression varies across studies, and a consensus definition that more explicitly defines adequate dosage and duration is needed to better assess patient outcomes as well as to translate such data into routine clinical practice (4). Identifying patients with treatment‐resistant depression is additionally complicated by the prevalence of nonadherence to treatment among patients with major depressive disorder, with nonadherence as high as 50% among those treated with antidepressants (5). Patients who undergo optimized antidepressant treatment and experience treatment failure may enter a complex multistep treatment approach involving switching to another antidepressant, adding another antidepressant, augmenting with another drug type (e.g., lithium or a second‐generation antipsychotic), or with cognitive and/or somatic therapy (e.g., electroconvulsive therapy or transcranial magnetic stimulation) (6).

Use of health care resources by patients with treatment‐resistant depression contributes significantly to the economic burden of the condition, which in the United States has been estimated to range from $29 to $48 billion annually (7). Two recent studies have compared health care costs among patients with treatment‐resistant and non–treatment‐resistant depression (8, 9). Amos et al., in a retrospective analysis of U.S. commercial claims data (2010–2015), found that patients with treatment‐resistant depression, compared with a matched sample of patients with non–treatment‐resistant depression, had on average 76% higher health care costs per patient per year ($17,261 vs. $9,790, respectively) (8). Olfson et al., in a study of Medicaid‐insured patients followed during the first year of antidepressant therapy, reported average health care costs for patients with treatment‐resistant depression that were 60% higher than those of patients with non–treatment‐resistant depression ($18,982 vs. $11,642) (9). In an earlier analysis of retrospective claims data (2001–2009) of patients with chronic major depressive disorder, defined as ≥2 years of continuous treatment, Olchanski et al. found that patients categorized as having treatment‐resistant depression had approximately 30% higher medical costs than those with chronic non–treatment‐resistant depression (10). In that study, treatment‐resistant depression was defined as having undergone at least four antidepressant trials (10), a more stringent definition of treatment resistance than that used in the latter studies of Amos et al. and Olfson et al., in which the definition was based on having failed two courses of antidepressant and/or augmentation therapy (8, 9). For real‐world studies using claims and/or hospital records data, it is essential to establish criteria for defining conditions in the absence of available diagnosis codes. The criteria may also need to be tailored to specific populations of interest, as in the Olchanski et al. study, in which outcomes of patients with chronic major depressive disorder were studied (10).

Further study of the clinical and economic burden of treatment‐resistant depression, especially among patients who have been hospitalized for an episode of major depressive disorder, is warranted to better characterize the risk of hospital readmission and associated hospital costs. Therefore, we conducted an all‐payer retrospective database analysis to characterize and evaluate hospital LOS, hospital costs, readmission risk, and the economic burden associated with readmissions among patients hospitalized for treatment‐resistant and non–treatment‐resistant major depressive disorder. Because this study was conducted from the hospital perspective, we defined treatment‐resistant depression on the basis of treatment information contained within the hospital records for each patient from admission to discharge.

## Methods

### Study Population

Adults (≥18 years of age) with a primary hospital discharge diagnosis of major depressive disorder were identified between January 1, 2012, and September 30, 2015, from the Premier Hospital Database (Charlotte, North Carolina). This data source is a nationally representative all‐payer database that captures data from >45 million hospital discharges from >600 acute‐care hospitals, representing approximately 20% of all U.S. hospital admissions. Data elements include demographic characteristics, hospital characteristics, procedures, and drug use information. Patient medical information available in the database is obtained from records collected at the hospital level for administrative purposes. The cost data recorded in the database reflect the cost to hospitals. Patient data in the database are de‐identified and thus in compliance with the Health Insurance Portability and Accountability Act.

The first hospital admission for major depressive disorder, on the basis of patients having a primary discharge diagnosis indicating major depressive disorder (*ICD‐9* codes: 296.2x, 296.3x; *ICD‐10* codes: F32.0–F32.5, F32.9, F33.0–F33.4x, F33.9), was defined as the index hospitalization with the corresponding admission date as the index date. Patients with major depressive disorder were stratified into two cohorts: those whose hospital treatment was suggestive of treatment‐resistant depression and those with non–treatment‐resistant depression. Patients were categorized as having treatment‐resistant depression if they had received electroconvulsive therapy, transcranial magnetic stimulation, vagus nerve stimulation, or olanzapine/fluoxetine capsules (Symbyax) at any time during their index hospitalization or if they received any antidepressant plus one of four Food and Drug Administration–approved antipsychotic medications for major depressive disorder (aripiprazole, brexpiprazole, olanzapine, quetiapine) during days 1–2 of the index hospitalization. The cohort with non–treatment‐resistant depression was composed of patients who did not receive any of the above listed treatments at any time during their index hospitalization. Because the Premier Hospital Database contains detailed information on the treatments and procedures administered during hospitalization but has limited data on prior outpatient history, the hospital treatment–based definition was used as criteria to establish treatment‐resistant depression status in this study.

### Patient and Index Hospitalization Characteristics

The evaluated patient demographic and clinical characteristics during the index hospital admission included age, gender, marital status, race, payer type, all patient refined diagnosis‐related group (APR‐DRG) severity level, APR‐DRG mortality level, major depressive disorder severity based on the *ICD‐9* or *ICD‐10* diagnosis codes at hospital discharge, and comorbid conditions. Other evaluated index hospitalization characteristics included year of index admission for major depressive disorder, admitting source, admission type, admitting physician specialty, hospital urban/rural status, hospital geographic region, hospital teaching status, hospital size, patient SI/SA status, and discharge status, and patient comorbidities.

### Propensity Score Matching (PSM)

PSM was used to balance differences in patient demographic and index hospitalization characteristics among the two cohorts. We generated propensity scores by using multivariable logistic regression analyses, in which age, gender, marital status, race, payer type, index hospitalization year, admitting source, admission type, admitting physician specialty, hospital urban/rural status, hospital geographic region, hospital teaching status, hospital bed size, discharge status, and select patient comorbid conditions were included as covariates. We conducted 1:1 matching of cohorts by using the nearest neighbor algorithm.

### Measured Outcomes

Average LOS and total hospital costs of the index hospitalization were determined for the treatment resistant and non–treatment‐resistant depression cohorts (both unmatched and matched patient groups). During a 6‐month follow‐up period (i.e., after discharge from the index hospitalization), the proportions of patients in the unmatched and matched study cohorts that had hospital readmissions (all‐cause, major depressive disorder–related, and related to suicidal ideation/suicidal attempt [SI/SA]) were evaluated. Major depressive disorder– and SI/SA‐related readmissions were based on *ICD‐9* or *ICD‐10* codes for these conditions in any position on the hospital discharge record. Additionally, the average total LOS for hospital readmissions and associated total readmission costs were determined as mean values for the study cohorts. Combined index hospitalization and readmission all‐cause costs and LOS were also calculated among patients in the matched cohorts. Those without readmission were assigned a LOS=0 and $0 cost.

### Statistical Analyses

For the unmatched and matched study cohorts of patients with treatment‐resistant depression and non–treatment‐resistant depression, we used bivariate descriptive statistics to describe patient demographic, patient characteristics, and index hospitalization characteristics. Hospital LOS and cost during the index admission, proportions of patients with hospital readmissions, and the associated LOS and costs during the 6‐month follow‐up period for the cohorts were also described by using bivariate descriptive statistics. The absolute differences and the relative percentage changes of the readmissions rates were assessed between the matched study cohorts. We conducted t tests and chi‐square tests to evaluate differences in continuous and categorical variables, respectively. We used multivariable Cox regression analyses to evaluate the impact of treatment‐resistant depression status on risk of hospital readmission at 1, 2, 3, and 6 months after discharge from the index hospitalization. A critical value of 0.05 was used to determine statistical significance. All statistical analyses were carried out using SAS version 9.4.

## Results

### Characteristics of Study Cohorts Prior to PSM

Prior to matching, 204,575 patients with a primary discharge diagnosis of major depressive disorder during the study period were identified. Among the hospitalized patients with major depressive disorder, 45,127 (22%) likely had treatment‐resistant depression according to our study definition; >90% were identified as having received an antidepressant in addition to an antipsychotic within their first 2 days of admission. Table 1 shows baseline demographic characteristics of the cohorts. Of the unmatched cohorts with treatment‐resistant depression and non–treatment‐resistant depression, mean age (45.9 vs. 43.1 years, p<0.001), proportion married (27.4% vs. 25.7%, p<0.001), race (white: 71.2% vs. 70.4%, p<0.001), and proportions with different payer types were significantly different. Table 2 shows patient characteristics at the index hospitalization. Among the unmatched patient cohorts, the comorbid conditions identified during the index hospitalization were generally more prevalent among patients with treatment‐resistant depression than among those with non–treatment‐resistant depression. Additionally, APR‐DRG severity (minor: 26.0% vs. 37.8%, p<0.001) and major depressive disorder severity (severe with or without psychotic behavior: 66.3% vs. 58.4%, p<0.001) differed for patients with treatment‐resistant and non–treatment‐resistant depression, respectively. Hospital characteristics also significantly differed among the cohorts (Table 2). Mean hospital LOS during the index hospitalization was 36% longer (7.4 vs. 5.4 days per patient, p<0.001) and mean hospital cost was 43% higher ($8,694 vs. $6,082 per patient, p<0.001) for patients with treatment‐resistant depression vs. those with non–treatment‐resistant depression, respectively (Table 2).

**Table 1:** Demographic characteristics at index hospitalization of unmatched and matched study cohorts^a^

	**Unmatched** ^b^				**Matched** ^c^			
	**TRD (N=45,127)**		**Non‐TRD MDD (N=159,448)**		**TRD (N=45,066)**		**Non‐TRD MDD (N=45,066)**	
**Variable**	**N**	**%**	**N**	**%**	**N**	**%**	**N**	**%**
Gender								
Female	25,627	56.8	90,179	56.6	25,587	56.8	25,512	56.6
Male	19,499	43.2	69,263	43.4	19,479	43.2	19,554	43.4
Marital status								
Married	12,351	27.4	40,955	25.7	12,338	27.4	12,243	27.2
Single	28,347	62.8	101,728	63.8	28,301	62.8	28,378	63.0
Other	4,303	9.5	16,420	10.3	4,301	9.5	4,320	9.6
Race								
White	32,132	71.2	112,209	70.4	32,086	71.2	32,106	71.2
Black	5,228	11.6	20,232	12.7	5,224	11.6	5,210	11.6
Other	7,694	17.1	26,620	16.7	7,683	17.1	7,683	17.1
Missing	73	0.2	387	0.2	73	0.2	67	0.2
Payer type								
Medicare	12,557	27.8	32,900	20.6	12,518	27.8	12,494	27.7
Medicaid	11,588	25.7	38,883	24.4	11,571	25.7	11,802	26.2
Managed care	11,361	25.2	41,445	26.0	11,356	25.2	11,385	25.3
Self‐pay	3,224	7.1	17,433	10.9	3,224	7.2	3,187	7.1
Commercial	2,910	6.5	12,145	7.6	2,910	6.5	2,825	6.3
Other	3,487	7.7	16,642	10.4	3,487	7.7	3,373	7.5

a MDD, major depressive disorder; TRD, treatment‐resistant depression.

b Age (mean±SD years): TRD=45.9±16.1; non‐TRD=43.1±16.4.

c Age (mean±SD years): TRD=45.9±16.1; non‐TRD=45.8±16.9. After propensity score matching, the patient cohorts were inspected to ensure they were well balanced with statistically similar key patient and hospitalization characteristics. All p>0.05.

**Table 2:** Index hospitalization characteristics of unmatched and matched study cohorts^a^

	**Unmatched**				**Matched** ^b^			
	**TRD (N=45,127)**		**Non‐TRD MDD (N=159,448)**		**TRD (N=45,066)**		**Non‐TRD MDD (N=45,066)**	
**Index hospitalization**	**N**	**%**	**N**	**%**	**N**	**%**	**N**	**%**
Admission year								
2012	13,901	30.8	45,664	28.6	13,883	30.8	13,885	30.8
2013	12,262	27.2	42,971	27.0	12,239	27.2	12,138	26.9
2014	11,103	24.6	41,116	25.8	11,092	24.6	11,092	24.6
2015	7,861	17.4	29,697	18.6	7,852	17.4	7,951	17.6
Admitting source								
Non‐health‐care facility	28,479	63.1	98,077	61.5	28,442	63.1	28,457	63.2
Transfer from different hospital	6,148	13.6	23,942	15.0	6,144	13.6	6,051	13.4
Transfer from detox unit	3,042	6.7	11,252	7.1	3,038	6.7	3,054	6.8
Transfer from health facility	2,648	5.9	8,568	5.4	2,642	5.9	2,689	6.0
Clinic	2,332	5.2	7,726	4.9	2,324	5.2	2,309	5.1
Court/law enforcement	642	1.4	3,781	2.4	642	1.4	629	1.4
Information not available	1,836	4.1	6,101	3.8	1,834	4.1	1,877	4.2
Admitting physician specialty								
Psychiatry	40,708	90.2	141,766	88.9	40,649	90.2	40,736	90.3
Internal medicine	448	1.0	2,621	1.6	448	1.0	427	1.0
General practice	279	.6	1,520	1.0	279	.6	244	.5
Other	3,692	8.2	13,541	8.5	3,690	8.2	3,659	8.1
Location status								
Urban	39,776	88.1	137,897	86.5	39,720	88.1	39,784	88.3
Rural	5,351	11.9	21,551	13.5	5,346	11.9	5,282	11.7
Geographic region								
South	19,363	42.9	76,824	48.2	19,352	42.9	19,230	42.7
Midwest	12,383	27.4	41,301	25.9	12,367	27.4	12,437	27.6
Northeast	7,998	17.7	22,924	14.4	7,972	17.7	7,993	17.7
West	5,383	11.9	18,399	11.5	5,375	11.9	5,406	12.0
Teaching status								
Yes	22,931	50.8	72,477	45.5	22,888	50.8	22,947	50.9
No	22,196	49.2	86,971	54.6	22,178	49.2	22,119	49.1
Bed size								
<300	12,011	26.6	47,112	29.6	12,001	26.6	11,898	26.4
≥300	33,116	73.4	112,336	70.5	33,065	73.4	33,168	73.6
Suicidal ideation/attempt	24,507	54.3	89,003	55.8	24,472	54.3	24,671	54.7
Discharge status								
Home	40,930	90.7	145,116	91.0	40,880	90.7	40,829	90.6
Transferred to facility	3,732	8.3	11,816	7.4	3,721	8.3	3,769	8.4
Deceased	7	.0	28	.0	7	.0	9	.0
Other	398	.9	2,260	1.4	398	.9	404	.9
Missing	60	.1	228	0.1	60	.1	55	.1
MDD severity^c^								
Mild	104	.2	742	.5	104	.2	206	.5
Moderate	2,723	6.0	14,801	9.3	2,720	6.0	3,823	8.5
Severe	29,909	66.3	93,128	58.4	29,873	66.3	26,665	59.2
Comorbidities^d^								
Substance abuse	24,490	54.3	89,888	56.4	24,460	54.3	24,556	54.5
Anxiety	19,595	43.4	62,970	39.5	19,552	43.4	19,447	43.2
Bipolar disorder	3,969	8.8	9,974	6.3	3,943	8.8	3,988	8.9
Attention disorder	1,428	3.2	5,527	3.5	1,428	3.2	1,447	3.2
Schizophrenia	1,844	4.1	3,379	2.1	1,800	4.0	1,720	3.8
Borderline personality	3,061	6.8	7,901	5.0	3,038	6.7	3,022	6.7
Posttraumatic stress disorder	5,856	13.0	16,101	10.1	5,826	12.9	5,827	12.9
Altered mental status	217	.5	919	.6	217	.5	207	.5
Pain	5,871	13.0	18,899	11.9	5,860	13.0	5,805	12.9
Hypertension	16,492	36.6	51,974	32.6	16,459	36.5	16,409	36.4
Asthma	4,941	11.0	14,991	9.4	4,925	10.9	4,893	10.9
Obesity	4,955	11.0	15,485	9.7	4,932	10.9	4,858	10.8
Hyperlipidemia	8,642	19.2	24,697	15.5	8,613	19.1	8,511	18.9
Type 2 diabetes	5,701	12.6	19,278	12.1	5,690	12.6	5,749	12.8
Esophageal reflux disease	7,489	16.6	21,520	13.5	7,457	16.6	7,357	16.3
Cardiovascular disease	4,814	10.7	15,610	9.8	4,806	10.7	4,764	10.6
Gastrointestinal disease	3,792	8.4	9,873	6.2	3,770	8.4	3,734	8.3
Skin lesions	5	.0	15	.0	5	.0	7	.0
Hypothyroidism	5,070	11.2	13,049	8.2	5,041	11.2	4,950	11.0
Hypokalemia	1,845	4.1	6,721	4.2	1,844	4.1	1,816	4.0
Urinary tract infection	2,388	5.3	7,373	4.6	2,379	5.3	2,290	5.1
Acute respiratory failure	19	.0	157	.1	19	.0	18	.0
Hospital LOS (days) (M±SD)	7.4±8.1		5.4±5.0		7.4±8.1		5.9±5.8	
Hospital cost ($) (M±SD)	8,694±11,866		6,082±7,494		8,681±11,818		6,632±8,948	

a LOS, length of stay; MDD, major depressive disorder; TRD, treatment‐resistant depression.

b After propensity score matching, the patient cohorts were inspected to ensure they were well balanced with statistically similar key patient and hospitalization characteristics. All p>0.05.

c MDD severity at discharge was categorized according to *ICD‐9* and *‐10* code descriptions.

d Comorbidities were identified using hospital discharge diagnosis records.

### Characteristics of *S*tudy *C*ohorts After PSM

After PSM, 45,066 patients were included in each matched cohort; patient characteristics did not significantly differ between cohorts; additionally, there were no significant differences in index hospitalization characteristics (Tables 1 and 2). The mean index hospitalization LOS remained significantly longer (7.4 vs. 5.9 days per patient, p<0.001; 26% longer) and mean hospital cost significantly higher ($8,681 vs. $6,632 per patient, p<0.001; 31% higher) for patients identified as having treatment‐resistant depression vs. those with non–treatment‐resistant depression, respectively (Table 2).

### Unmatched Study Cohorts: Frequency of Hospital Readmissions and Associated LOS and Costs

Prior to matching, the proportions of patients with all‐cause (24.4% vs. 18.4%, p<0.001), major depressive disorder–related (17.0% vs. 12.1%, p<0.001), and SI/SA‐related (12.8% vs. 9.0%, p<0.001) readmissions in the 6‐month follow‐up period were greater among those with treatment‐resistant depression vs. those with non–treatment‐resistant depression.

Furthermore, among unmatched cohorts, the mean LOS for all‐cause, major depressive disorder–related, and SI/SA‐related hospital readmissions during the 6‐month follow‐up were 57% (3.3 vs. 2.1 days per patient, p<0.001), 67% (2.2 vs. 1.3 days per patient, p<0.001), and 68% (1.5 vs. 0.9 days per patient, p<0.001) longer, respectively, for patients with treatment‐resistant depression vs. those with non–treatment‐resistant depression. Mean total hospital costs for all‐cause ($3,697 vs. $2,434 per patient, p<0.001), major depressive disorder–related ($2,318 vs. $1,377 per patient, p<0.001), and SI/SA‐related ($1,491 vs. $901 per patient, p<0.001) readmissions were 52%, 68%, and 65% higher, respectively, for patients with treatment‐resistant depression vs. those with non–treatment‐resistant depression.

### Matched Cohorts: Frequency of Hospital Readmissions and Associated LOS and Costs

After matching, the proportions of patients with all‐cause (24.4% vs. 20.0%, p<0.001), major depressive disorder–related (17.0% vs. 13.3%, p<0.001), and SI/SA‐related (12.8% vs. 9.5%, p<0.001) readmissions during the 6‐month follow‐up period were greater among patients with treatment‐resistant depression vs. those with non–treatment‐resistant depression (Table 3). The differences in readmission rates corresponded to relative increases in the rates of all‐cause (21.9%), major depressive disorder–related (28.2%), and SI/SA‐related (35.0%) readmissions for patients with treatment‐resistant depression vs. those with non–treatment‐resistant depression during the 6‐month follow‐up (Table 3). Cumulative monthly percentages of patients readmitted to the hospital during the 6‐month follow‐up are also shown in Table 3.

**Table 3:** Cumulative percentages of readmissions for matched study cohorts during the 6 months following discharge from index hospitalization^a^

		**Non‐TRD**		
	**TRD**	**MDD**		**Relative**
	**(N=45,066)**	**(N=45,066)**	**Difference** ^b^	**Increase** ^c^
**Readmission**	**%**	**%**	**%**	**%**
All‐cause readmission				
0–1 month	10.8	8.9	1.9	21.0
0–2 months	15.2	12.4	2.8	22.6
0–3 months	18.2	14.9	3.3	22.3
0–4 months	20.5	16.7	3.8	22.8
0–5 months	22.4	18.2	4.2	23.2
0–6 months	24.4	20.0	4.4	21.9
MDD‐related readmission				
0–1 month	7.5	6.1	1.3	21.7
0–2 months	10.6	8.4	2.2	26.5
0–3 months	12.7	10.0	2.7	27.1
0–4 months	14.3	11.1	3.2	28.4
0–5 months	15.6	12.1	3.5	28.9
0–6 months	17.0	13.3	3.7	28.2
SI/SA‐related readmission				
0–1 month	4.9	3.9	1.0	26.3
0–2 months	7.4	5.5	1.9	34.4
0–3 months	9.3	6.8	2.4	35.9
0–4 months	10.6	7.8	2.8	35.9
0–5 months	11.7	8.6	3.1	36.3
0–6 months	12.8	9.5	3.3	35.0

a MDD, major depressive disorder; SI/SA, suicidal ideation/suicidal attempt; TRD, treatment‐resistant depression.

b Difference: Percentage of cohort with TRD minus percentage of cohort without TRD.

c Relative increase: (percentage of cohort with TRD minus percentage of cohort without TRD)/percentage of cohort without TRD.

Cox regression results showed that compared with matched patients with non–treatment‐resistant depression, patients with treatment‐resistant depression had a 25% increased risk for all‐cause readmission, a 31% increased risk for major depressive disorder–related readmission, and a 37% increased risk for SI/SA‐related readmission during the 6‐month follow‐up (Table 4). These results were consistent for readmissions within 1, 2, and 3 months postdischarge from the index hospitalization (Table 4).

**Table 4:** Risk for hospital readmission within 1, 2, 3, and 6 months of discharge from index hospitalization for matched study cohorts^a^

	**TRD vs. non‐TRD MDD**	
**Readmission**	**Hazard ratio**	**95% CI**
All‐cause readmission		
0–1 month	1.22	1.17–1.27
0–2 months	1.24	1.20–1.28
0–3 months	1.24	1.20–1.28
0–6 months	1.25	1.22–1.28
MDD‐related readmission		
0–1 month	1.23	1.17–1.29
0–2 months	1.28	1.22–1.33
0–3 months	1.29	1.24–1.34
0–6 months	1.31	1.26–1.35
SI/SA‐related readmission		
0–1 month	1.28	1.20–1.36
0–2 months	1.35	1.28–1.42
0–3 months	1.37	1.31–1.44
0–6 months	1.37	1.32–1.43

a MDD, major depressive disorder; SI/SA, suicidal ideation/suicidal attempt; TRD, treatment‐resistant depression. All p values <0.001.

**Figure 1:**
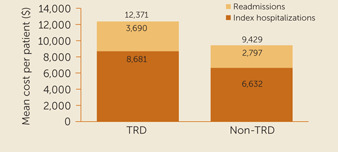
Mean total all‐cause costs per patient with major depressive disorder for index hospitalization and readmissions 6 months postdischarge^a^

After matching, the mean LOS for all‐cause, major depressive disorder–related, and SI/SA‐related hospital readmissions during the 6‐month follow‐up were 38% (3.3 vs. 2.4 days per patient, p<0.001), 45% (2.2 vs. 1.5 days per patient, p<0.001), and 52% (1.5 vs. 1.0 days per patient, p<0.001) longer, respectively, for patients with treatment‐resistant vs. those with non–treatment‐resistant depression. Mean total hospital costs for all‐cause ($3,690 vs. $2,797 per patient, p<0.001), major depressive disorder–related ($2,314 vs. $1,608 per patient, p<0.001), and SI/SA‐related ($1,489 vs. $998 per patient, p<0.001) readmissions were 32%, 44%, and 49% higher, respectively, for patients with treatment‐resistant depression vs. those with non–treatment‐resistant depression (Figure 1). The combined (index hospitalization and all‐cause readmissions) mean all‐cause hospital LOS (10.7 vs. 8.3 days per patient, p<0.001; 30% longer) and hospital costs ($12,371 vs. $9,429 per patient, p<0.001; 31% greater) were also significantly greater for patients with treatment‐resistant depression vs. those with non–treatment‐resistant depression (Figure 1).

a

## Discussion

To our knowledge, this analysis of >90,000 patients hospitalized with an acute episode of major depressive disorder who were matched for similar patient and hospitalization characteristics constitutes the first study of the incremental burden of treatment‐resistant depression from a hospitalist's perspective, for whom costs and readmission risk are of significant concern. By using a practical clinical treatment algorithm to identify patients whose depression was treatment resistant, we found that the cost of the index hospitalization for major depressive disorder was 31% higher for patients with treatment‐resistant depression compared with those with non–treatment‐resistant depression. Additionally, treatment‐resistant depression was associated with substantially higher risk for hospital readmission, which represented a significant incremental economic burden compared with patients whose depression was not treatment resistant. When the analyses were controlled for patient and hospitalization differences by using PSM, we found that those with treatment‐resistant depression had a 25% increased risk for all‐cause readmission compared with patients with non–treatment‐resistant depression; when type of readmission was examined, there was a 31% increased risk for major depressive disorder–related readmission and a 37% increased risk for SI/SA‐related readmission within 6 months of discharge. The increased readmission rates of patients with treatment‐resistant depression corresponded with an increase in hospitalization costs ranging from 32% to 49%, compared with patients with non–treatment‐resistant depression. These results are noteworthy because non–treatment‐resistant major depressive disorder is already widely recognized as a considerable health care and economic burden. In fact, a recent study of 39,155 patients admitted to an acute care inpatient facility for serious medical conditions (e.g., acute myocardial infarction, pneumonia), showed that comorbid serious mental illness was associated with a 28% increased risk for readmission within 30 days (11).

Because comorbidity was controlled for in our analysis, as well as differences in hospitalization characteristics, reasons for the added risk for rehospitalization among patients with treatment‐resistant depression are likely related to persistent depression symptoms and greater symptom severity. This relationship is reflected in our findings showing that most all‐cause readmissions and costs were attributed to major depressive disorder– and SI/SA‐related readmissions. Furthermore, on the basis of the *ICD* code description at the index hospitalization, we found that major depressive disorder was categorized as severe among a greater proportion of patients with treatment‐resistant depression compared with patients with non–treatment‐resistant depression, which is consistent with the understanding that patients whose depression is treatment resistant may have more severe depression than patients whose depression is not treatment resistant. However, this observation must be interpreted with caution when considering the limited availability of the *ICD* code descriptions for major depressive disorder severity (i.e., nearly one‐third of the study population had no specific description of major depressive disorder severity). Further study aimed at differentiating severity of major depressive disorder among patients with treatment‐resistant depression may be warranted; access to outpatient records, which were not available in the data source used for this study, may be useful in this context. Of note, this analysis was restricted to patients with an inpatient stay, and thus the assessments of the incremental burden of treatment‐resistant depression were not sensitive to the increased risk for initial hospitalization among patients with the condition (8, 9, 12).

According to our hospital treatment‐based definition, 22% of the overall study population were considered to have treatment‐resistant depression, a prevalence that is within the range reported in other studies (16%–26%) in which the criteria used to define the condition differed (8, 9). Approximately one‐quarter of patients with treatment‐resistant depression and one‐fifth of patients with non–treatment‐resistant depression were readmitted to the hospital within 6 months of their initial discharge. The higher rate of readmission for patients with treatment‐resistant depression was observed even after controlling for discharge status. For both groups, nearly half of all readmissions occurred within the first month after discharge. These data highlight the potential benefit of care continuity and providing timely follow‐up after hospitalization for an episode of major depressive disorder. Because approximately 90% of all patients with a primary discharge diagnosis of major depressive disorder were discharged to their home, perhaps a more effective transition to outpatient care may reduce readmission rates and the associated cost burden among this patient population.

Another finding of this study was the significant added risk for suicide associated with treatment‐resistant depression among patients with major depressive disorder, which has been shown in prior studies among other populations (7, 8). However, this is the first analysis to confirm this association in perhaps the more severe cases of patients hospitalized for the disorder; we found that patients whose depression was treatment resistant had nearly a 40% greater chance of having a SI/SA‐related readmission compared with patients whose depression was not treatment resistant. Suicide is a prominent and growing problem in the United States, with the Centers for Disease Control and Prevention reporting it as the tenth leading cause of death (13). Identifying those patients at greatest risk for such an adverse outcome is critical for providing appropriate interventions.

Because patients' complete medical histories (e.g., treatments prescribed in the outpatient setting) were not available in the Premier Hospital Database, we used the type of treatment received during hospitalization as a proxy to determine treatment‐resistant depression status, which was not confirmed by clinical documentation. Most patients (>90%) with treatment‐resistant depression were identified as having received an antipsychotic in addition to an antidepressant within their first 2 days of admission. In a separate, not yet published, retrospective analysis of claims data, we defined treatment‐resistant depression as having completed at least two antidepressant and/or antipsychotic regimens of adequate dosage and duration, similar to the most commonly used criteria for treatment‐resistant depression reported by the AHRQ (4) and to the definition used in other recent analyses of claims data (8, 9). In our unpublished analysis, we found that patients with treatment‐resistant depression had approximately sevenfold greater usage of antidepressant polypharmacy and approximately fourfold greater usage of atypical antipsychotics compared with patients whose major depressive disorder was not treatment resistant. Thus, within the limitations of the current hospital‐based study, the definition we used aligns well with our recent observations of treatment patterns of patients with treatment‐resistant depression, as well as with commonly used treatment algorithms for treatment‐resistant depression (6, 14), and contributes to the clinical research of patients with treatment‐resistant depression in the inpatient setting. However, further research is warranted on the identification of patients with treatment‐resistant depression on the basis of data recorded during hospitalization.

Inpatient treatments, such as electroconvulsive therapy, are more expensive than antidepressants and antipsychotics, and because a majority of patients with treatment‐resistant depression were identified as having received an antidepressant and an antipsychotic, the incremental costs observed in this study are less likely to be inflated compared with patients with non–treatment‐resistant depression. Because hospital readmissions data were captured only when patients were readmitted into the Premier Hospital system, the absolute rate of hospital readmissions may have been underestimated. However, it is likely that the relative risks of readmissions between the patient cohorts were generally consistent. The patient matching process further reduced the measured differences between the cohorts and provided a more conservative estimate of differences in readmissions. Although the follow‐up period was only 6 months, this period was likely sufficient to capture readmission data, because nearly half of all readmissions occurred within the first month after discharge.

The Premier Hospital Database did not include data on outpatient coverage and services. Thus, this analysis did not include key outpatient intermediary outcomes that can affect readmission measures, which may have led to underestimation of the economic burden related to hospitalizations of these patients. At least 600 hospitals across the United States contribute data to Premier; however, these results may not be generalizable to the entire population of U.S. patients with major depressive disorder or to the entire U.S. population hospitalized for major depressive disorder. Because of limitations in the database, the reasons for hospitalizations, treatment decisions, readmissions, etc., could not be established, and further study using other data sources with such information may be warranted. On the other hand, this data source is nationally representative and frequently used to evaluate characteristics of hospitalizations in multiple therapeutic areas, including mental illness (15, 16), and findings from such a database are generally considered valuable (17).

## Conclusions

After the analyses were controlled for differences in patient and hospitalization characteristics, we found that hospitalized patients with treatment‐resistant depression had greater risk for hospital readmission and were more likely to incur greater hospitalization costs than patients with non–treatment‐resistant depression. Improving the transition of care to the outpatient setting as well as providing more effective treatment and care continuity may help reduce the personal and hospital burden of patients with treatment‐resistant depression who are hospitalized. Future research of the impact of such interventions is warranted.

## Footnotes

a Δ=$2,941, p<0.001. All costs were calculated among all patients; patients with no readmission were assigned a readmission cost of $0. TRD, treatment‐resistant depression.
